# Sensorimotor learning during synchronous speech is modulated by the acoustics of the other voice

**DOI:** 10.3758/s13423-024-02536-x

**Published:** 2024-07-02

**Authors:** Abigail R. Bradshaw, Emma D. Wheeler, Carolyn McGettigan, Daniel R. Lametti

**Affiliations:** 1 MRC Cognition and Brain Sciences Unit, University of Cambridge, Cambridge, UK; 2 Department of Speech, Hearing and Phonetic Sciences, University College London, London, UK; 3 Department of Psychology, Acadia University, Wolfville, Nova Scotia Canada

**Keywords:** Sensorimotor learning, Speech production, Speech perception

## Abstract

This study tested the hypothesis that speaking with other voices can influence sensorimotor predictions of one’s own voice. Real-time manipulations of auditory feedback were used to drive sensorimotor adaptation in speech, while participants spoke sentences in synchrony with another voice, a task known to induce implicit imitation (phonetic convergence). The acoustic-phonetic properties of the other voice were manipulated between groups, such that convergence with it would either oppose (incongruent group, *n = 15*) or align with (congruent group, *n = 16*) speech motor adaptation. As predicted, significantly greater adaptation was seen in the congruent compared to the incongruent group. This suggests the use of shared sensory targets in speech for predicting the sensory outcomes of both the actions of others (speech perception) and the actions of the self (speech production). This finding has important implications for wider theories of shared predictive mechanisms across perception and action, such as active inference.

**Supplementary Information:**

The online version contains supplementary material available at 10.3758/s13423-024-02536-x.

## Introduction

Speech production is a complex sensorimotor act, relying on the integration of top-down predictions with bottom-up sensory input from the sound of our voice. These top-down predictions are thought to consist of sensory targets for the expected/intended auditory and somatosensory consequences of a given speech movement (Guenther, 2016; Parrell & Houde, 2019). Comparison of such targets with the actual auditory and somatosensory feedback generated by a speech movement can allow the system to detect and correct any deviations from these targets (i.e., prediction error) to ensure that our speech productions remain accurate and finely tuned (Parrell, Lammert, et al., 2019a; Parrell & Houde, 2019; Tourville & Guenther, 2011). Evidence for the use of such targets during speech motor control comes from sensory feedback perturbation paradigms, in which real-time auditory or somatosensory feedback during speaking is artificially changed (Burnett et al., 1998; Houde & Jordan, 1998; Tremblay et al., 2003). For example, the spectral properties of a produced vowel sound can be manipulated such that the resonant frequencies (known as formants) are shifted closer to a different vowel category, for example, alteration of the first and second formants in an utterance of the word ‘head’ can make it sound more like ‘had’. When repeatedly exposed to such changes, speakers are found to unconsciously adjust the way they produce speech sounds to compensate for this apparent deviation from their auditory target; this adjustment is known as *speech motor adaptation*. Crucially, such changes are found to persist for a period after the perturbation of auditory feedback is removed (Purcell & Munhall, 2006), suggesting that a recalibration process has occurred to reflect the new mapping between motor commands and sensory consequences.

Two types of sensorimotor learning are thus important for the acquisition and maintenance of speech: (1) learning of the sensory targets associated with particular speech sounds, and (2) learning sensorimotor mappings so that the system knows what motor commands achieve those sensory targets. According to models of speech motor control, the formation of sensory targets for speech happens early in development, starting with auditory targets learnt using speech input from other talkers during infancy (Guenther, 2016). However, once auditory targets have been acquired, these models do not currently consider any ongoing role of perception of other voices in shaping speech auditory targets across the lifespan. This contrasts with findings from a historically separate sociolinguistics literature showing that interactions with other voices – the central reason we speak – can have rapid and lasting effects on our productions of speech sounds, such that we start to sound more similar to those other voices (Pardo et al., 2017). This ‘vocal convergence’ is seen both at higher levels such as in speakers’ use of semantics and vocabulary (Garrod & Anderson, 1987) and at lower levels in terms of the acoustic-phonetic properties of their voices (Aubanel & Nguyen, 2020; Bradshaw & McGettigan, 2021; Garnier et al., 2013).

Although traditionally viewed as a strategy employed to achieve social attunement among speakers (Giles et al., 1991), more recently a lower-level sensorimotor mechanism account of vocal convergence has been proposed, in which perception of the other voice triggers an updating of the auditory targets used to control the speaker’s own speech movements (Sato et al., 2013; Späth et al., 2022). This idea of shared targets across speech production and perception is consistent with recent proposals of shared mechanisms of prediction across the two domains (Friston et al., 2021; Pickering & Gambi, 2018; Pickering & Garrod, 2013). However, the vast majority of research using sensory feedback perturbation involves studying speakers producing speech on their own. A few exceptions exist in the literature, whose findings suggest that sensorimotor learning during the production of single words can be affected by perceptual experience of other voices (Bourguignon et al., 2016; Lametti et al., 2014; Shiller & Rochon, 2014). None of these studies, however, involved more naturalistic sentence-level speech, or considered vocal convergence and its potential relationship to speech motor adaptation.

In a previous study, we investigated speech motor adaptation during an interactive speaking task, in which speakers synchronised the timing of their productions of sentences with another voice (the accompanist) whilst their own speech formants were altered in real-time (Bradshaw et al., 2023). Such synchronous speech is found across a variety of everyday contexts, such as in places of worship and sports stadiums (Cummins, 2018). Compared to a group who spoke alone, adaptation responses during such synchronous speech showed increased variability across individual speakers. An exploratory analysis suggested that some of this variability may relate to the congruency between the production changes required for each speaker to converge to the other voice and those required for adaptation to the formant perturbation. However, the design of this study made it difficult to disentangle the potential effects of vocal convergence from other aspects of the synchronous speech task, such as changes in speaking rate and attention.

**Fig. 1:**
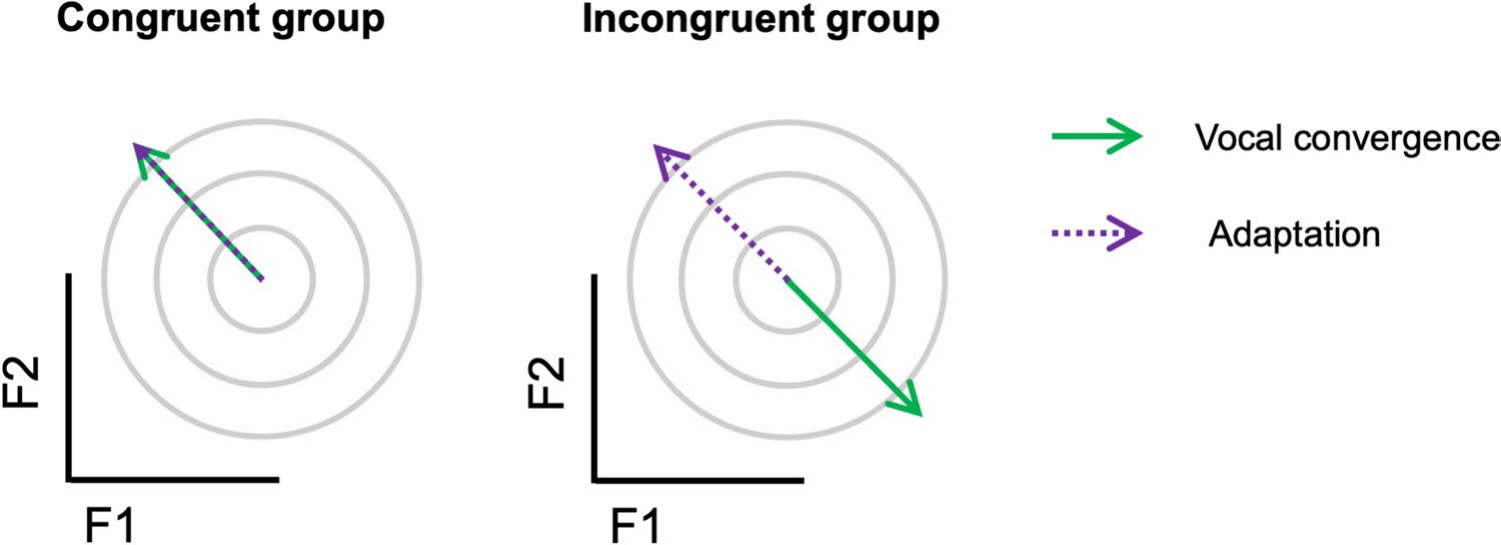
Illustration of the experimental manipulation. For the congruent group, adaptation and vocal convergence responses should pull formants in the same direction, whereas for the incongruent group these would be pulled in opposite directions. Note that both groups experienced an upwards perturbation of F1 and a downwards perturbation of F2, applied to their own speech in near-real time (see Methods*: Apparatus*)

In the current study, we aimed to test this hypothesis more directly; namely, that the effect of synchronous speech on formant changes during speech motor adaptation will depend on the congruency of formant changes induced by a simultaneous vocal convergence process. This was designed to test the wider claim that speech perception and speech production operate on shared underlying auditory targets, that is, that vocal convergence is driven by an updating of the auditory targets that are used for sensorimotor error correction with one’s own speech feedback. To test this, we compared adaptation between two groups who both performed a synchronous speech task but with different accompanist voices. The acoustic-phonetic properties of these two voices were manipulated in order to explicitly vary the congruency between the direction of formant change required for convergence to the other voice, and the direction of formant change required for adaptation to the formant perturbation (see Fig. 1). The two conditions were thus perfectly matched on all aspects of the task, except the relationship between convergence and adaptation. We predicted that if the auditory targets used for adaptation are changed through vocal convergence, we should see significantly reduced adaptation in an incongruent voice condition (in which convergence and adaptation are pulling formants in opposite directions) compared to a congruent voice condition (in which these are pulling in the same direction). Conversely, if speech motor adaptation and the auditory targets it operates with are somehow shielded from concurrent vocal convergence, we would expect adaptation to be identical across the two conditions.

## Methods

### Open Practices Statement

The design, hypotheses and analyses for this experiment were pre-registered prior to collection of data on the Open Science Framework (https://osf.io/gkjes). Pseudonymised data and analysis code are also available on this platform (https://osf.io/h26ur/).

### Participants

A total of 41 participants were recruited for this experiment. Data from six participants had to be excluded due to a technical fault with the auditory equipment. A further four participants were excluded because they had baseline formants that did not relate to the accompanist voice formants as intended (i.e., F1 was not higher/F2 was not lower than accompanist in the congruent group or vice versa in the incongruent group). This left a total of 31 participants whose data were analysed, with 16 in the congruent group and 15 in the incongruent group. This total is one more than our pre-registered target sample size (30), as the congruent voice version of the experiment was run with one additional participant than planned; the decision to keep this participant’s data was made prior to any analysis. This sample size was chosen based on previous work in which significant sensorimotor adaptation in response to feedback perturbations is seen at the group level in groups of eight to 12 participants (Houde & Jordan, 1998; Lametti et al., 2018, p. 20).

All participants were biologically female native speakers of Canadian English (mean age = 21.5 years, *SD* = 5.17), with no reported history of speech, language or reading problems and no history of hearing loss. Female participants were recruited from the Acadia University community via the Psychology Department’s online participant recruitment website; the study was approved by the Acadia University Research Ethics Committee.

### Procedure

**Fig. 2:**
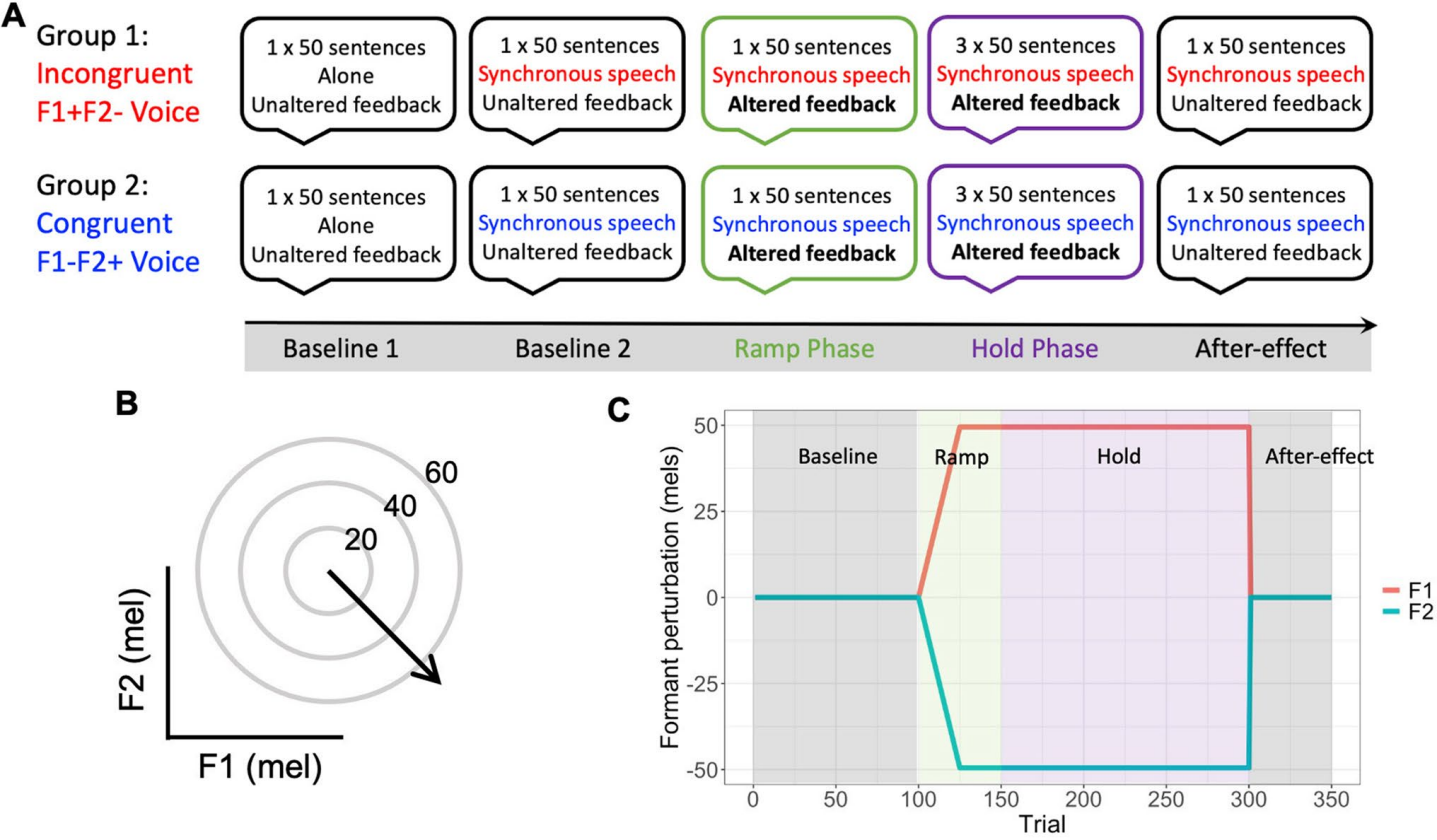
Experimental procedure. (**A**) Procedure of the experiment for the two groups: the incongruent voice condition and the congruent voice condition. (**B**) Schematic representation of the formant perturbation used for the ramp and hold phases. (**C**) Plot shows the implementation of the formant perturbation across trials. Shading indicates phase as labelled (baseline, ramp, hold and after-effect)

The procedure for the experiment is illustrated in Fig. 2A. Participants were instructed to read aloud sentences as they were presented on a computer screen. Throughout the experiment, the sound of their voice was played back to them in real time through headphones (either unaltered or altered). A set of 50 sentences was presented once in each of seven blocks, giving a total of 350 trials per participant (with sentence order randomised across blocks). In the first block, participants were told to read the sentences normally without exposure to the accompanist voice. From block 2 onwards the pre-recorded accompanist voice was played through headphones (in addition to the participant’s own voice) and participants were asked to read the sentences in synchrony with this voice. The voice stimuli used were manipulated in a between-groups design (see Stimuli). Prior to starting the first block, participants were given ten practice trials of solo speech to familiarise themselves with the task. Participants were then given an additional ten practice trials of synchronous speech prior to starting block 2. Between each of the following blocks, participants were allowed to take a short break (e.g., to drink some water), and instructed to indicate through silent gesture when they were ready to start the next block (through a thumbs up). These breaks were generally around 15–30 s long.

Across all blocks of the experiment, each trial began with the visual presentation of the sentence to be read on the computer screen and a three-click countdown played through the headphones (with an interstimulus interval of 1 s between clicks). For blocks 2–7 this was followed by presentation of the pre-recorded accompanist voice producing the same sentence. For block 1, participants were told to simply read aloud the sentence after the clicks had finished. From block 2 onwards they were told to synchronise the timing of their speech with that of the accompanist voice, using the countdown to help them start at the same time as the accompanist. The total duration of each trial was 8.5 s. The total duration of each testing session was about 1 h.

### Stimuli

The 50 sentences used for the task were taken from the Harvard IEEE corpus of sentences (IEEE Subcommittee on Subjective Measurements, 1969). The accompanist voice stimuli consisted of recordings of a female speaker of Canadian English reading the 50 sentences. This speaker was instructed to read the sentences at a regular conversational volume and speed, and to enunciate each word clearly. These recordings were then altered using the MATLAB-based software programme Audapter (Cai, 2015; Cai et al., 2008) to provide two stimulus sets for the two conditions: stimuli for the incongruent group were created by shifting F1 up and F2 down with a joint perturbation magnitude of 120 mels at an angle of 325° (resulting in an average F1 of 641 mels and F2 of 1303 mels); stimuli for the congruent group were created by shifting F1 down and F2 up with a joint perturbation magnitude of 190 mels at an angle of 100° (resulting in an average F1 of 524 mels and F2 of 1,435 mels). To ensure that the altered voices were outside the typical F1–F2 range for this population, the parameters used for shifting the accompanist voice were based on a sample of baseline formant frequencies from female speakers of Canadian English recorded in two prior studies (Lametti et al., 2023; Shiller et al., 2023). Convergence to each of the two accompanist voices would thus require changes in formants in opposite directions; an increase in F1 and decrease in F2 in the incongruent group, but the reverse in the congruent group.

As previously mentioned, participants were screened based on their baseline (block 1) formants and excluded if these did not relate to the accompanist voice of their allotted condition as intended. Participants in the congruent group had a mean baseline F1 of 623.89 mels (*SD* = 30.1) and a mean baseline F2 of 1368.31 mels (*SD* = 41.61); participants in the incongruent group had a mean baseline F1 of 593.63 mels (*SD* = 29.98) and a mean baseline F2 of 1339.06 mels (*SD* = 24.50). Taking the absolute difference between each group’s baseline formants and those of their respective accompanists, this distance was significantly greater in the congruent than the incongruent group for both F1 (congruent mean = 99.74, incongruent mean = 47.79, *t *= 4.81, *df* = 28.9, *p* < .001) and F2 (congruent mean = 66.71, incongruent mean = 36.12, *t *= 2.51, *sd* = 24.55, *p *= .019).

### Apparatus

Participants spoke into a head-mounted microphone (Shure, WH20) and heard their own voice through headphones (Sennheiser, HD 280 Pro). A laptop computer (Dell), mixer (Behringer), audio interface (RME Babyface Pro), and the MATLAB-based program Audapter (Cai et al., 2008) were used to record and manipulate the sound of the voice. Participants’ speech was presented at approximately 70 dB SPL (varying dynamically with changes in the amplitude of the participant’s voice) mixed with speech-shaped masking noise presented at 60 dB SPL; this was used to mitigate participants’ perception of their own unaltered voice either via air-conducted or bone-conducted feedback. The accompanist voice was presented at about 65 dB SPL, to approximate natural conditions in which speech from another talker is heard at a lower level compared to self-produced speech. An LED VU metre (American Audio, DB Display MKII) provided visual feedback to participants about their speech volume; this was calibrated such that lights on the metre turned green for speech input of about 70dB SPL, and red or yellow for speech significantly higher or lower (respectively) than this volume. The total feedback loop latency of the audio set-up was measured using methods outlined in Kim et al. (2020). Using a value of 3 for the nDelay parameter within Audapter, the total feedback delay associated with both hardware and software latencies for perturbed speech feedback was measured at 12 ms. This latency is far below the delay levels that have been reported to disrupt speech adaptation (Max & Maffett, 2015; Shiller et al., 2020).

To implement our formant perturbations, an openly available MATLAB-based application was used (*Audapter,* Cai, 2015; Cai et al., 2008). Speech was recorded at a sampling rate of 48 kHz (down-sampled to 16 kHz) with a buffer size of 96 samples. The same perturbation of the first and second formants was used for both groups, specifically an upwards shift in F1 and a downwards shift in F2, with a joint magnitude of 70 mels resulting in a shift in each formant of 49.5 mels (see Fig. 2B). Adaptation to this formant perturbation would thus require a decrease in F1 and an increase in F2. As illustrated in Fig. 2C, the feedback perturbation was ramped up across the first 25 trials of block 3 (the ramp phase), before being held constant until the end of block 6 (hold phase). The perturbation was then removed completely for the final block to assess the after-effects of speech motor learning (after-effect phase).

### Acoustic analysis

Formant frequencies in the recordings of the participants’ speech were analysed using a custom-made script in Praat (Boersma & Weenink, 2021). The script first isolates the vocalised portions of the speech signal using Praat’s autocorrelation method (Boersma, 1993). F1 and F2 values in Hertz were then extracted using a Linear Predictive Coding (LPC) approach. Formant frequencies were then converted into mels and averaged across each sentence by taking the mean.

### Quantification of adaptation

To quantify participants’ responses to the formant perturbation, we first calculated a production change measure for each formant separately. Each of the 50 produced formant frequencies in block 6 (the final adaptation block) were normed to the F1/F2 frequencies for block 2 (baseline) on a sentence-by-sentence basis; the average of these values was then taken to give an average production change value for each participant (for each formant). Adaptation was then calculated by quantifying the extent to which these changes in produced formant frequencies directly countered the direction of our perturbation in F1–F2 space (Lametti et al., 2018; Niziolek & Guenther, 2013, p. 20). First, the inverse of the vector corresponding to the perturbation in F1–F2 space was found; this represents the direction of perfect adaptation. Next, this inverse vector was compared to a vector representing the participant’s own changes in F1 and F2 (relative to block 2); the angular difference between these vectors was taken and then the cosine of this difference multiplied by the magnitude of production change. This results in a consistent scale for both F1 and F2 changes in which positive values indicate formant changes that opposed the direction of the perturbation, and negative values indicate changes that followed the direction of the perturbation. This measure was calculated for each trial following the introduction of the perturbation and then averaged within each block (for blocks 3–7).

### Hypotheses

Our main hypothesis of interest for this experiment was that participants in the incongruent group would show significantly reduced adaptation and after-effects compared to participants in the congruent group. We also predicted that prior to experience of the formant perturbation, participants in both groups would show evidence of convergence in their formants towards those of the accompanist voice they experienced, in the form of convergent changes in the F1 and F2 of their speech productions during the second baseline block (first block with synchronous speech) relative to the first baseline block (solo speech). We further predicted that these convergent changes in block 2 would be in the same direction as subsequent adaptation for the congruent group, but in the opposite direction to adaptation in the incongruent group. Lastly, we predicted that the extent to which these convergent changes agreed with the direction of adaptation would be positively correlated with the magnitude of the adaptation response across the whole sample.

All of the above hypotheses were pre-registered. A detailed break-down of the pre-registered statistical analyses run to test these hypotheses (including full model structure for linear mixed effects models) are given in the Online Supplementary Material (OSM) S1. For the sake of brevity, these are reported in a more concise manner in the text below.

## Results

### Convergence responses

**Fig. 3:**
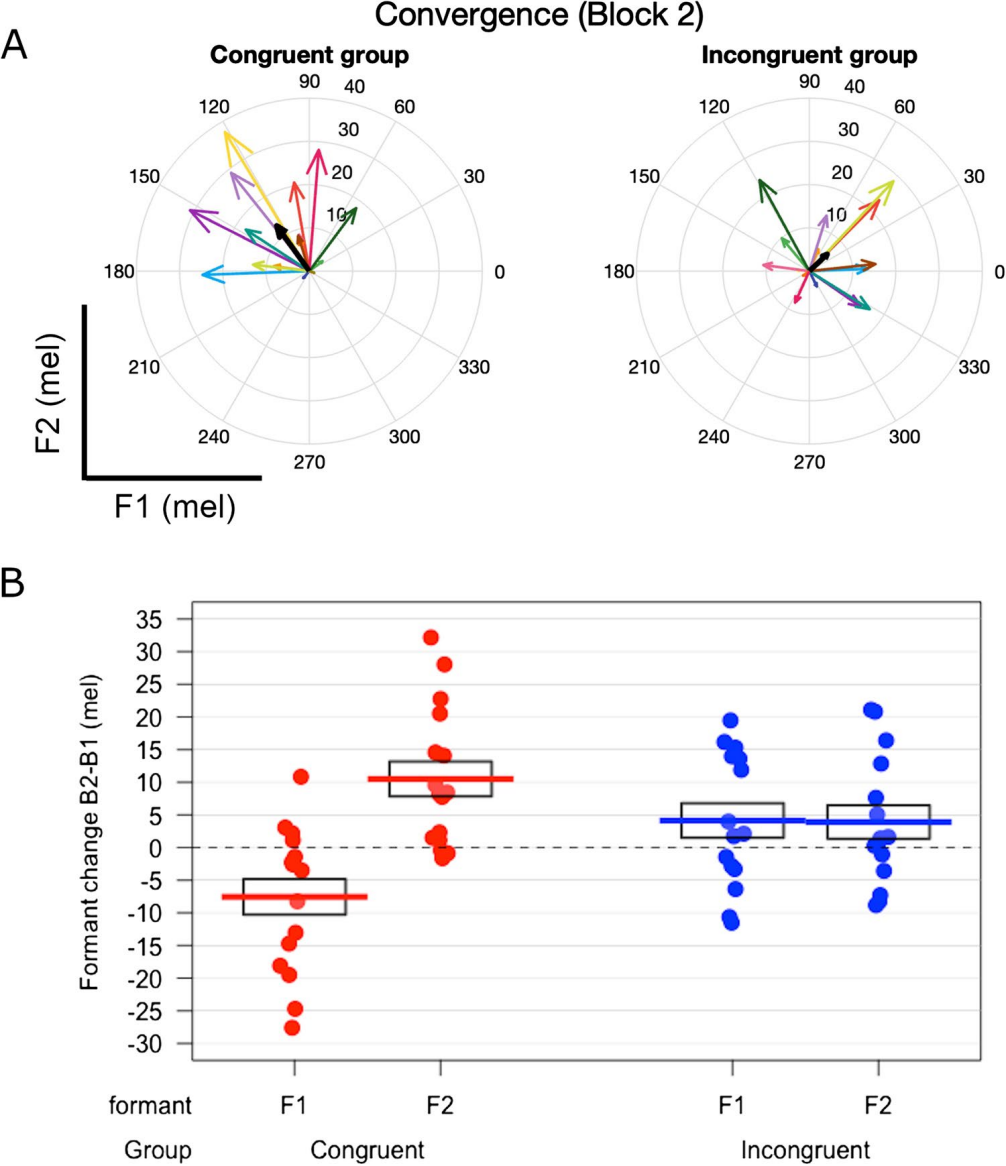
Vocal convergence in F1 and F2. (**A**) Changes in F1 and F2 from block 1 to block 2 are represented as vectors in F1–F2 space, to illustrate convergence-adaptation congruency. Coloured arrows indicate individual participant changes, thick black arrows indicate group averages. (**B**) F1 and F2 changes from block 1 to block 2 in mels in the two accompanist voice conditions. Dashed line indicates zero

Changes in formant frequencies from block 1 to block 2 are illustrated for the two groups in Fig. 3. The congruent voice group showed significant convergence to the accompanist voice in the form of a significant decrease in F1 (*β* = -7.55, *t*(35.58) = -2.69, *p* = .011) but a significant increase in F2 (*β* = 10.51, *t*(46.42) = 3.51, *p* = .001) (linear mixed modelling analysis). Conversely, neither F1 nor F2 changes were significantly different from zero in the incongruent voice group.

### Adaptation responses

**Fig. 4:**
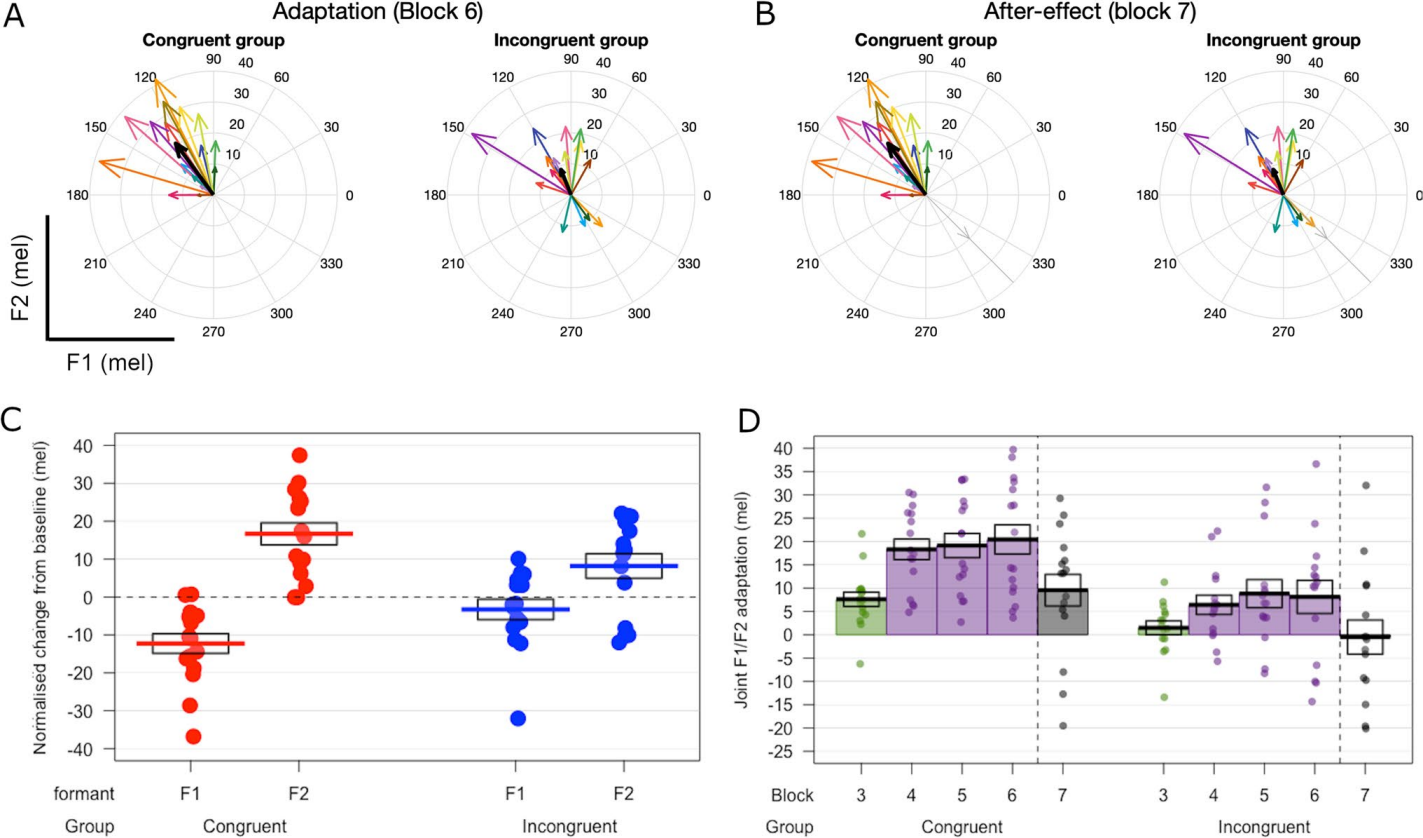
Speech motor adaptation during synchronous speech. (**A**) Thin coloured arrows indicate adaptation responses for each participant in the form of vectors in F1/F2 space (for block 6). Group averages are shown in thick black arrows. The light grey arrow at 315° indicates the direction of the formant perturbation. (**B**) Equivalent vectors for the after-effects of adaptation in block 7. (**C**) Change in produced formant frequencies from baseline block 2 to the final block of perturbed feedback (block 6). Dots indicate individual participant averages, thick lines indicate group means and boxes show standard errors. (**D**) Adaptation responses for blocks 3–7. Colour coding of bars indicates phase: green shows the ramp phase (formant perturbation gradually increased), purple shows the hold phase (perturbation held constant), and black shows the after-effect phase (perturbation removed). Dotted vertical lines indicate removal of the feedback perturbation for block 7

Figure 4 illustrates vectors representing individual adaptation (A) and after-effects (B) across the two groups. Figure 4C illustrates changes in produced formants from block 2 to block 6. As can be seen, participants in the congruent group showed a significant decrease in produced F1 (*t*(15) = -4.73, *p* < .001) but a significant increase in F2 from block 2 to block 6 (*t*(15) = 5.76, *p* < .001), indicating significant adaptation (one-sample two-sided t-tests). Conversely, the incongruent group showed a significant increase in F2 (*t*(14) = 2.53, *p* = .024), but no significant change in F1. Figure 4D plots adaptation (quantified as the component of formant changes that directly countered the perturbation) across blocks 3–7 of the experiment in the two groups. For each participant, a one-sample two-sided t-test was run to test if adaptation was significantly greater than zero (significant adaptation), significantly lower than zero (significant ‘following’ response) or not significantly different from zero. The number of participants in each of these categories is shown for the two groups in Table 1.

**Table 1:** Frequency of participants showing adaptation/following in the two groups

	Congruent group	Incongruent group
**Significant adaptation response**	13	10
**Significant following response**	0	3
**No significant adaptation**	3	2

Figure 4D shows that the overall shapes of the distributions plotting adaptation across blocks are similar across the two groups (but at different overall magnitudes); that is, both groups show a large increase from block 3 (ramp phase) to block 4 (hold phase), followed by smaller increases across subsequent blocks in the hold phase, and then finally a decrease for block 7 (after-effect phase). A linear mixed-modelling analysis found a significant interaction between block (3–7) and group on adaptation (*χ*^*2*^(4) = 10.79, *p* = .029), in which the effect of block on adaptation was significantly greater in the congruent group than the incongruent group; that is, the congruent group showed a greater build-up of adaptation with increasing experience of the formant perturbation. By the final block with perturbed feedback, the incongruent group showed significantly smaller adaptation than the congruent group (*β* = -12.34, *t*(41) = -3.67, *p* = .001), as well as significantly reduced after-effects of adaptation once the perturbation was removed (*β* = -10.05, *t*(41)= -10.05, *p* = .005).

To relate convergence to subsequent adaptation, we calculated a measure of adaptation-convergence congruency that quantifies to what extent the direction of formant changes shown by participants from block 1 to block 2 agreed with the direction of perfect adaptation to the subsequent perturbation. As predicted, a one-sided independent-samples t-test found that this measure was significantly greater in the congruent group (*M* = 12.77) compared to the incongruent group (*M* = -0.16): *t*(28.89) = -3.35, *p* = .001). However, no significant correlation was found between this measure and the magnitude of the subsequent adaptation response (measured relative to block 2).

## Discussion

As predicted, this study found significantly reduced speech motor adaptation when simultaneous vocal convergence opposed the direction of adaptation (incongruent voice group) compared to when vocal convergence was in the same direction as adaptation (congruent voice group). This difference in adaptation cannot be attributed to anything other than the difference in the acoustics of the accompanist voice between the conditions, since all other aspects of the task were identical. This comparison thus controls for any potential effects of auditory masking, changes in speaking rate, and attention related to performance of the synchronous speech task. Overall, this demonstrates that the magnitude of the measured adaptation response to a formant perturbation depends on the acoustics of other voices being interacted with. This provides support for an intimate relationship between mechanisms of speech perception for others’ voices and production of one’s own voice, an aspect that is currently not incorporated into dominant models of speech motor control (Guenther, 2016; Houde & Nagarajan, 2011; Parrell, Ramanarayanan et al., 2019b).

One can question, however, the precise mechanism underlying this effect of perception of other voices on speech motor adaptation. One interpretation could be that convergence involves an updating of internal acoustic targets for speech that are then used in subsequent speech motor learning. This would thus view the current results as evidence for shared sensory targets across speech perception and production. An alternative interpretation, however, might argue that the group difference in adaptation could be driven by a reflexive mimicry response to the accompanist voice that is simply summed with the compensatory adaptation response, without any offline updating of statistical relationships between acoustic parameters (i.e., formants) and linguistic targets (i.e., speech sounds). These accounts thus differ in whether such effects of vocal convergence on adaptation should persist when the acoustic input from the other voice is removed. Since the accompanist voice was always present throughout the adaptation and after-effect phases, it is arguably difficult to tease apart these two possibilities. However, there are several patterns worth highlighting in the current findings that may favour one interpretation over the other.

For the congruent voice group, we found clear evidence of vocal convergence in both F1 and F2 in block 2, prior to introduction of the feedback perturbation in block 3. Adaptation then appeared to operate relative to this new starting position, with participants showing significant and robust adaptation when measured relative to block 2. This pattern of results is most readily explained by assuming that convergence involves an updating of sensory targets for speech that are then used for speech motor learning with the formant perturbation. If convergence did not change such internal targets, the effect of convergent changes in formants (themselves driven by some independent mechanism, e.g., mimicry) would be to coincidentally compensate for the current formant perturbation, thus lowering prediction error and removing the need for further compensatory changes. Conversely, we observed strong and robust adaptation in this group even when taking into account changes in formants caused by convergence (i.e., when measuring formant changes relative to block 2). This suggests that convergence is thus underpinned by an updating of auditory targets for speech, such that the perturbation has the expected effect of generating prediction errors that are corrected for, on top of any changes relating to convergence.

Participants in the incongruent voice group did not show evidence of convergence to the accompanist voice before the perturbation was introduced. Previous research suggests that participants can raise their F1 and decrease their F2 (as required for convergence to the incongruent voice) just as easily as the reverse modification in response to formant perturbations during sentence level speech (Lametti et al., 2018; Shiller et al., 2023). However, the distance between the average baseline (block 1) formants of each group and the formants of their respective accompanist voices was significantly smaller in the incongruent compared to the congruent group; this may therefore explain the difference in convergence. It is interesting to note, however, that our previous study investigating the effect of synchronous speech on adaptation (without manipulation of accompanist formants) found significant convergence in a sample with baseline formants at a similarly small distance to the accompanist voice formants (Bradshaw et al., 2023).

Nevertheless, exposure to the accompanist voice clearly restricted the incongruent group’s subsequent adaptation to the formant perturbation. This suggests that, despite the lack of an initial convergence response, participants in this group did update their internal speech targets to be closer to the accompanist voice. In this case, paradoxically the effect of the perturbation would have been to bring the participant’s speech productions closer to their new acoustic target, resulting in reduced prediction error and thus limited changes in produced formant frequencies. For some individuals, however, significant convergence was achieved by the end of the perturbation phase, reflected in apparent ‘following’ responses in which formants were moved in the same direction as the perturbation. To our knowledge, across all previous studies of adaptation during solo sentence production (three studies totalling 180 adaptation sessions), only two followers have been reported (Bradshaw et al., 2023; Lametti et al., 2018; Shiller et al., 2023). The observation here of three followers for a condition performed by a group of 15 participants is thus notable. This suggests that, at least in some cases, convergence can ‘win out’ over adaptation.

This interpretation in terms of shared sensory targets across perception and production is further supported by recent evidence reporting transfer of passive perceptual statistical learning into production. Murphy et al. (2023) exposed participants to a series of minimal pair utterances (beer-pier) in which the statistical relationship between fundamental frequency (F0) and voice onset time (VOT) was either typical for English (higher F0s and longer VOTs for pier vs. beer) or reversed (lower F0s and longer VOTs for pier vs. beer). Participants were then prompted to repeat one of the minimal pair items themselves; crucially, however, the test stimuli used to elicit such productions were identical across typical and reversed conditions. Nevertheless, they found that participants previously exposed to the reversed condition showed a convergent down-weighting of F0 in their own productions of the minimal pairs, relative to the typical condition. In this study, this group difference in production cannot be attributed to any kind of reflexive mimicry response that relies on the physical characteristics of the item used to cue production; instead, this effect was attributed to an offline updating of statistical relationships between acoustic parameters and linguistic targets in perception that is transferred over to production. Similar evidence was found by Sato et al. (2013), who reported that convergence to another voice during a syllable repetition task transferred into an ‘after-effects’ phase in which the same syllables were visually (instead of acoustically) cued. They argued that vocal convergence thus involves adaptive plasticity in sensory targets, in which perceptual learning with another voice can transfer to self-voice production in an offline fashion. Together with the results of the current study, this evidence thus argues against a reflexive mimicry mechanism and in favour of an offline perceptual learning account of vocal convergence.

A limitation of the current work is that the paradigm of synchronous speech employed is not representative of conversational speech. Synchronous speech is, however, a naturalistic behaviour found across a variety of real-world speaking contexts, such as places of worship, schools, sports stadiums, and protest marches (Cummins, 2018). Further, for our experimental purposes, synchronous speech provides an interesting context in which speech feedback from the self-voice and from another voice is received concurrently, forcing the parallel processing of both input streams at the same time. This allows us to study the dynamics of interactions between vocal convergence and speech motor adaptation when these processes must operate simultaneously, placing particularly high demands on the self-monitoring system to perform accurate attribution of speech feedback to self versus other. Nevertheless, there is evidence for the operation of each process in more everyday speaking contexts; vocal convergence is observed during natural conversations (Pardo, 2006; Pardo et al., 2018), while speech motor adaptation has been demonstrated during natural production of variable sentences (Lametti et al., 2018). It would thus be of interest to replicate the current study design in the context of a conversational task, ideally with live interactions between interlocutors. This would allow the study of interactions between these processes in the context of a sequential turn-taking task that places fewer demands on self-monitoring. This would further allow for the manipulation of higher-level social factors such as the relationship or power-dynamic between the two interlocutors, which have been shown to affect the extent of vocal convergence (Bourhis & Giles, 1977; Gregory & Webster, 1996; Michalsky & Schoormann, 2017). The demonstration that such social factors can affect the relative weighting of adaptation for speech motor control would be a theoretically significant finding, and place this lower-level phenomenon in the wider context of communicative and interactive speech.

Overall, this study suggests that vocal convergence and speech motor adaptation operate on the same internal speech targets; that is, contrary to assumptions in the speech motor control literature, the acoustic targets that control speech productions are not static, but remain to a certain degree flexible in response to experience of other voices across the lifespan. Overall, this suggests that mechanisms of prediction and prediction error calculation may overlap across speech perception and production (Pickering & Garrod, 2013; Skipper et al., 2017). This is consistent with wider theories of the relationship between action and perception such as predictive coding and active inference, which assume a common sensory prediction mechanism and computational neural architecture for both processes (Adams et al., 2013; Friston, 2011). It would be fruitful for future research to continue to test the predictions of these wider theories more directly in the context of speech, perhaps our most inherently sensorimotor behaviour. In particular, it will be of interest to consider how a potential parity in computations across production and perception can be achieved in tandem with accurate source monitoring.

## Supplementary Information

Below is the link to the electronic supplementary material.

Supplementary file1 (DOCX 336 KB)

## Data Availability

Anonymised data reported on in this article are available on the Open Science Framework (https://osf.io/h26ur/).

## Code availability

The code used to perform all analyses reported on in this manuscript is available on the Open Science Framework (https://osf.io/h26ur/).
