# Correction: Sensorimotor learning during synchronous speech is modulated by the acoustics of the other voice

**DOI:** 10.3758/s13423-025-02651-3

**Published:** 2025-03-13

**Authors:** Abigail R. Bradshaw, Emma D. Wheeler, Carolyn McGettigan, Daniel R. Lametti

**Affiliations:** 1 MRC Cognition and Brain Sciences Unit, University of Cambridge, Cambridge, UK; 2 Department of Psychology, Acadia University, Wolfville, Nova Scotia Canada; 3 Department of Speech, Hearing and Phonetic Sciences, University College London, London, UK

**Correction: Psychonomic Bulletin & Review**

10.3758/s13423-024-02536-x

**Figure:**
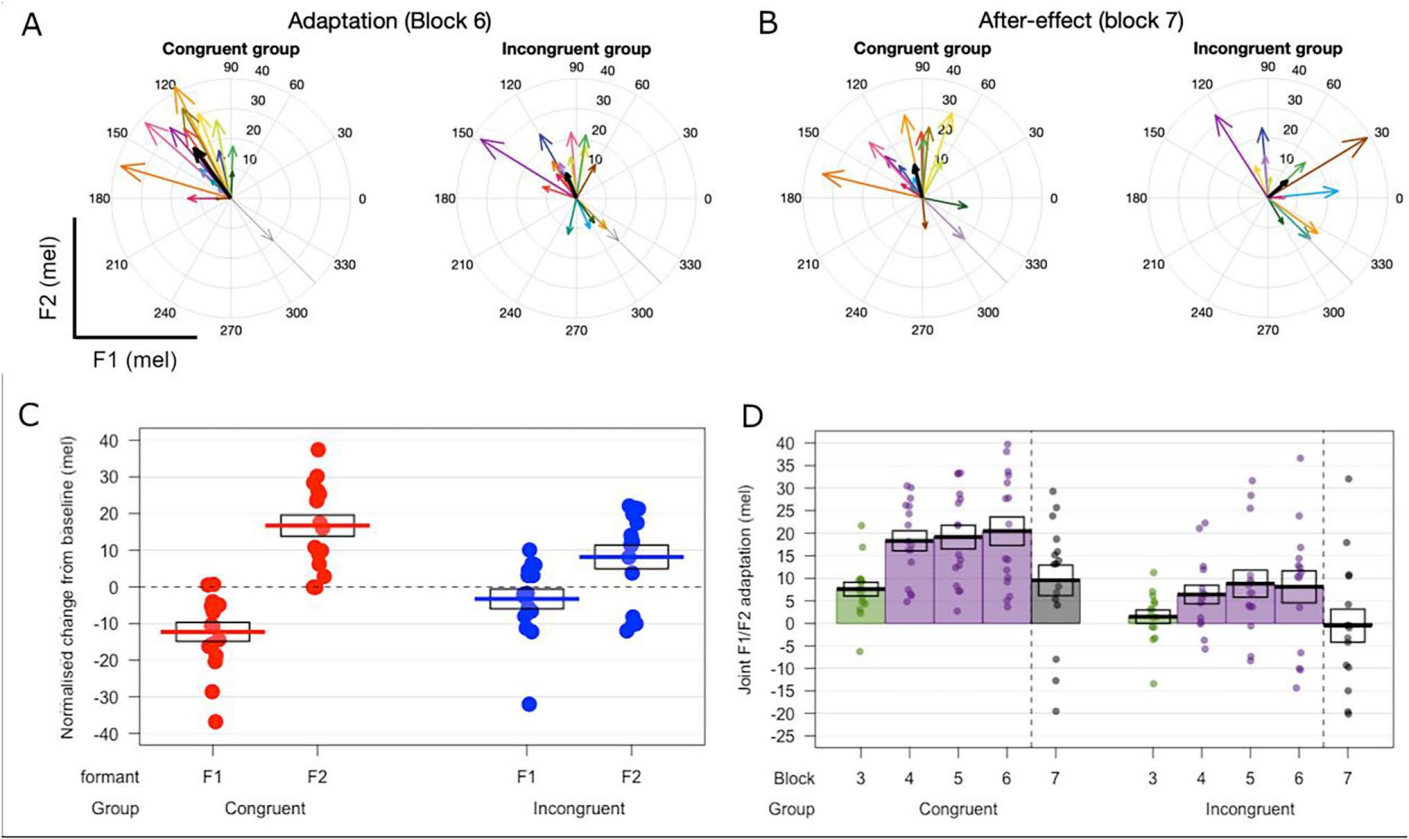
**Fig. 4**. Speech motor adaptation during synchronous speech. (**A**) Thin coloured arrows indicate adaptation responses foreach participant in the form of vectors in F1/F2 space (for block 6). Group averages are shown in thick black arrows. Thelight grey arrow at 315° indicates the direction of the formant perturbation. (**B**) Equivalent vectors for the after-effects ofadaptation in block 7. (**C**) Change in produced formant frequencies from baseline block 2 to the final block of perturbedfeedback (block 6). Dots indicate individual participant averages, thick lines indicate group means and boxes showstandard errors. (**D**) Adaptation responses for blocks 3–7. Colour coding of bars indicates phase: green shows the rampphase (formant perturbation gradually increased), purple shows the hold phase (perturbation held constant), and blackshows the after-effect phase (perturbation removed). Dotted vertical lines indicate removal of the feedback perturbationfor block 7

This paper published with an incorrect Figure 4, the correct figure is presented here.

