# Supplementary material for: Sensorimotor learning during synchronous speech is modulated by the acoustics of the other voice
Source: Psychon Bull Rev. 2024 Jul 2;32(1):306–16. doi: 10.3758/s13423-024-02536-x (PMC11836077; doi:10.3758/s13423-024-02536-x)
Supplement: Supplementary file 1 — Supplementary file1 (DOCX 336 KB) [file 13423_2024_2536_MOESM1_ESM.docx]

**Supplementary Material S1: Detailed reporting of pre-registered statistical analyses**

**Pre-registered hypotheses**

***Hypothesis 1:*** Participants who synchronise their speech with a voice whose formants have been shifted in the same direction as the formant perturbation will show significantly reduced adaptation compared to participants who synchronise their speech with a voice whose formants have been shifted in the opposite direction to the formant perturbation. This is because in the former group convergence and adaptation will be in opposite directions (incongruent condition), whereas in the latter group convergence will be in the same direction as adaptation (congruent condition).

***Hypothesis 2:*** Participants in the congruent group will show a significant adaptation response at the group level. Participants in the incongruent group may or may not show significant adaptation at the group level.

***Hypothesis 3:*** Participants in both groups will show evidence of convergence in their formants towards those of the accompanist voice in the form of convergent changes in F1 and F2 during the second baseline block when the synchronous speech task is introduced.

***Hypothesis 4:*** Changes in formants between blocks 1 and 2 (convergence) will be in the same direction as adaptation for the congruent group but in the opposite direction to adaptation in the incongruent group. The extent to which these convergent changes agree with the direction of adaptation will be positively correlated with adaptation responses across the whole sample.

**Results**

*Adaptation responses*

Figure S1 illustrates vectors representing individual adaptation (A) and after-effects (B) across the two groups. Figure S1D plots adaptation (quantified as the component of formant changes that directly countered the perturbation) across blocks 3 to 7 of the experiment in the two groups. To test for significant adaptation to the formant perturbation in individual participants, one-sample two-sided t-tests were used to test if adaptation in block 6 was significantly different from zero. This found significant adaptation (adaptation significantly greater than zero) in 13 out of 16 participants in the congruent group and 10 out of 15 participants in the incongruent group. The remaining three participants in the congruent group and two of the participants in the incongruent group showed no significant adaptation. A further three participants in the incongruent group showed adaptation responses that were significantly lower than zero; these negative values indicate that formants were instead moved in the same direction as the perturbation, typically termed a ‘following’ response.

To determine if adaptation was significant at the group level in each of our two groups, we ran two-sided one-sample t-tests for F1 and F2 production changes (from block 2 to block 6) separately (see Figure S1C). For the congruent group, these t-tests found a significant decrease in F1 (*t*(15) = -4.73, *p* < .001) but a significant increase in F2 (*t*(15) = 5.76, *p* < .001). For the incongruent group, these t-tests found a significant increase in F2 (*t*(14) = 2.53, *p* = .024), but no significant change in F1 (*t*(14) = -1.22, *p* = .243). These results thus support Hypothesis 2.

**
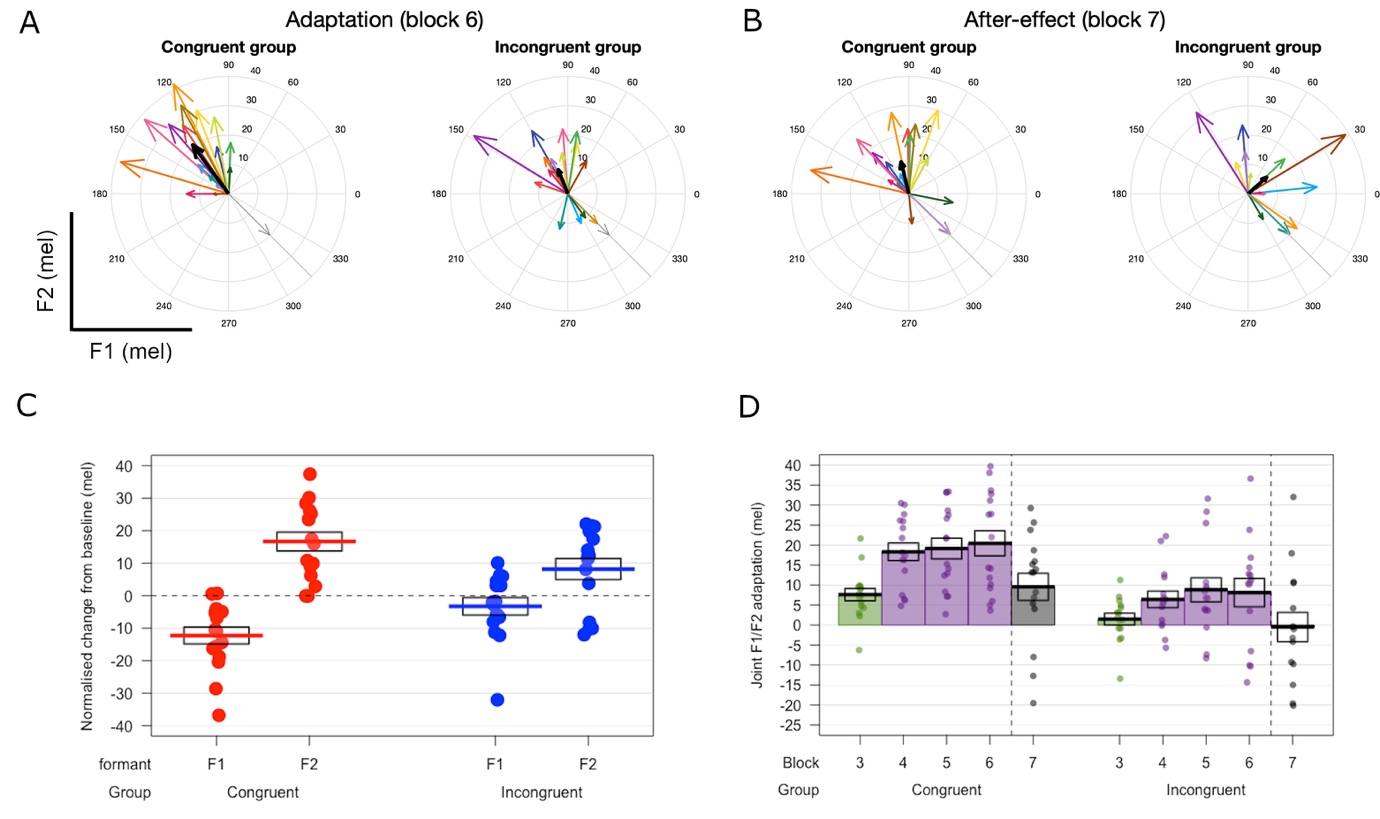
*Figure S1:*** ***Speech motor adaptation during synchronous speech.*** *(A) Thin coloured arrows indicate adaptation responses for each participant in the form of vectors in F1/F2 space (for block 6). Group averages are shown in thick black arrows. The light grey arrow at 315 degrees indicates the direction of the formant perturbation. (B) Equivalent vectors for the after-effects of adaptation in block 7. (C) Change in produced formant frequencies from baseline block 2 to the final block of perturbed feedback (block 6). Dots indicate individual participant averages, thick lines indicate group means and boxes show standard errors. (D) Adaptation responses for blocks 3 to 7. Colour coding of bars indicates phase: green shows the ramp phase (formant perturbation gradually increased), purple shows the hold phase (perturbation held constant), and black shows the after-effect phase (perturbation removed). Dotted vertical lines indicate removal of the feedback perturbation for block 7.*

To compare adaptation between groups, we used a linear mixed modelling analysis to model the fixed effects of group (congruent versus incongruent) and block (3, 4, 5, 6 and 7) on adaptation. Our models also included random intercepts of participant and sentence (random slopes of block by participant and block by sentence were not included due to failures of model convergence). A likelihood ratio test was used to compare two models, one in which block and group had additive effects, and one in which block and group had interactive effects. This found that the interactive model provided a better fit to the data than the additive model *χ^2^*(4) = 10.79, *p* = .029. Taking block 3 and the incongruent group as the reference conditions, this interactive model found that the change from block 3 was significantly greater in the congruent than the incongruent group for block 4 (*β* = 5.79, *t*(7660.99) = 2.73, *p* = .006), block 5 (*β* = 4.21, *t*(7660.99) = 1.99, *p* = .047), and block 6 (*β* = 6.22, *t*(7660.99) = 2.94, *p* = .003), but not for block 7. Planned follow-up contrasts were then performed on the interactive model to investigate (a) the effect of block in each group and (b) the effect of group for blocks 6 and 7. These found that both groups showed a significant difference between block 3 and blocks 4, 5 and 6 and between block 7 and blocks 4, 5 and 6 (p < .02 in all cases, using the Tukey method for adjusting for multiple comparisons). There were however no significant differences between blocks 3 and 7, 4 and 5, 4 and 6 or 5 and 6. Furthermore, a significant effect of group was found for both block 6 (*β* = -12.34, *t*(41) = -3.67, *p* = .001) and block 7 (*β* = -10.05, *t*(41)= -10.05, *p* = .005). These results thus support Hypothesis 1, that adaptation and after-effects would be significantly reduced in the incongruent group compared to the congruent group.

*Vocal convergence responses*

Changes in formant frequencies from block 1 to block 2 are illustrated for the two groups in Figure S2. To test for evidence of vocal convergence in participant’s formants in the two groups, LMM analyses were run on participant’s formant frequencies during blocks 1 and 2, for F1 and F2 separately. All models included fixed effects of block (1 or 2) and group (congruent versus incongruent), random intercepts of sentence and participant, and random slopes of block by participant and block by sentence. We predicted a significant interaction between block and group, in which the effect of block on F1 and F2 frequencies will be different according to the accompanist voice experienced. For F1, a likelihood ratio test found that a model containing an interaction between these fixed effects indeed provided a better fit to the data than an additive model (*χ^2^*(1) = 8.69, *p* = .003). This interactive model found a significant interaction between block and group (*β* = -11.69, *t*(28.99) = -3.07, *p* = .005), in which the change in F1 from block 1 to 2 was significantly lower (more negative) in the congruent than the incongruent group, as expected. For F2, a likelihood ratio test found that the interactive model did not provide a better fit to the data (*χ^2^*(1) = 3.18, *p* = .075). With block 1 and incongruent as the reference conditions, the additive model found a significant effect of block (*β* = 7.32, *t*(38.97) = 3.26, *p* = .002) and a significant effect of group (*β* = 30.87, *t*(29) = 2.50, *p* = .018). This absence of a significant interaction reflects the unexpected increase in F2 shown in the incongruent group. This analysis was followed up with a non-preregistered analysis in order to test whether the changes in F1 and F2 from block 1 to block 2 were significantly different from zero in both groups. To do this, we ran zero-intercept LMMs for each formant separately on the change in frequency from block 1 to block 2; these models contained a fixed effect of group, and random intercepts of participant and sentence. These found that while the congruent group showed evidence of significant convergence to the accompanist voice in the form of a significant decrease in F1 (*β* = -7.55, *t*(35.58) = -2.69, *p* = .011) but significant increase in F2 (*β* = 10.51, *t*(46.42) = 3.51, *p* = .001), neither F1 nor F2 changes were significantly different from zero in the incongruent group. Therefore, hypothesis 3 was only partly supported, in that we only found significant convergence in the congruent but not the incongruent group.

To relate convergence to subsequent adaptation, we calculated a measure of adaptation-convergence congruency that quantifies to what extent the direction of formant changes shown by participants from block 1 to block 2 agreed with the direction of perfect adaptation to the subsequent perturbation. This measure is calculated using the same method as for adaptation, but substituting changes in formants from block 2 to block 6 with changes from block 1 to block 2. Vectors representing these formant changes are illustrated in Figure S2A. As predicted, a one-sided independent samples t-test found that this measure was significantly greater in the congruent group (*M* = 12.77) compared to the incongruent group (*M* = -0.16): *t*(28.89) = -3.35, *p* = .001). However, no significant correlation was found between this measure and the magnitude of the subsequent adaptation response. These results thus only partially support Hypothesis 4.


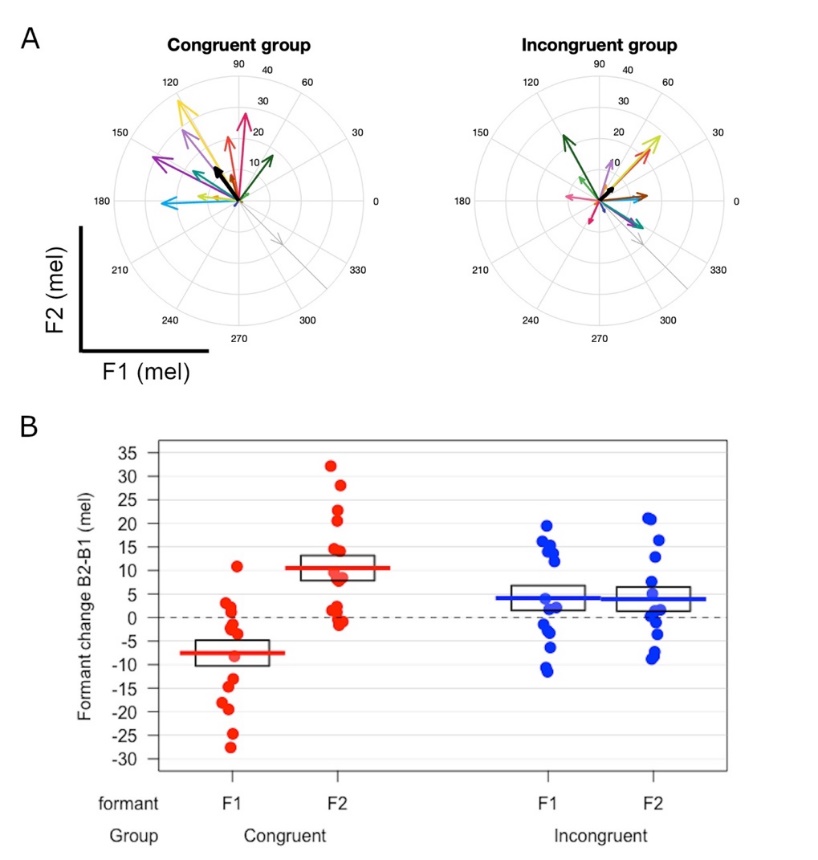


***Figure S2:*** ***Vocal convergence in F1 and F2.*** *(A) Changes in F1 and F2 from block 1 to block 2 are represented as vectors in F1-F2 space, to illustrate convergence-adaptation congruency. Coloured arrows indicate individual participant changes, thick black arrows indicate group averages. (B) F1 and F2 changes from block 1 to block 2 in mels in the two accompanist voice conditions. Dashed line indicates zero.*
